# *Bitis arietans* Snake Venom and Kn-Ba, a Snake Venom Serine Protease, Induce the Production of Inflammatory Mediators in THP-1 Macrophages

**DOI:** 10.3390/toxins13120906

**Published:** 2021-12-16

**Authors:** Ângela Alice Amadeu Megale, Fabio Carlos Magnoli, Felipe Raimondi Guidolin, Kemily Stephanie Godoi, Fernanda Calheta Vieira Portaro, Wilmar Dias-da-Silva

**Affiliations:** 1 Immunochemistry Laboratory, Butantan Institute, São Paulo 05503-900, Brazil; fabio.magnoli@butantan.gov.br (F.C.M.); guidolin.felipe@gmail.com (F.R.G.); kemily.godoi@esib.butantan.gov.br (K.S.G.); 2 Laboratory of Structure and Function of Biomolecules, Butantan Institute, São Paulo 05503-900, Brazil

**Keywords:** *Bitis arietans* venom (BaV), Kn-Ba, inflammation, cytokines and chemokines, PGE_2_, THP-1 macrophages

## Abstract

*Bitis arietans* is a snake of medical importance found throughout sub-Saharan Africa and in savannas and pastures of Morocco and western Arabia. The effects of its venom are characterized by local and systemic alterations, such as inflammation and cardiovascular and hemostatic disturbances, which can lead to victims’ death or permanent disability. To better characterize the inflammatory process induced by this snake’s venom, the participation of eicosanoids and PAF (platelet- activating factor) in this response were demonstrated in a previous study. In addition, edema and early increased vascular permeability followed by an accumulation of polymorphonuclear (PMN) cells in the peritoneal cavity were accompanied by the production of the eicosanoids LTB_4_, LTC_4_, TXB_2_, and PGE_2_, and local and systemic production of IL-6 and MCP-1. In this context, the present study focused on the identification of inflammatory mediators produced by human macrophages derived from THP-1 cells in response to *Bitis arietans* venom (BaV), and Kn-Ba, a serine protease purified from this venom. Here, we show that Kn-Ba, and even the less intensive BaV, induced the production of the cytokine TNF and the chemokines RANTES and IL-8. Only Kn-Ba was able to induce the production of IL-6, MCP-1, and IP-10, whereas PGE_2_ was produced only in response to BaV. Finally, the release of IL-1β in culture supernatants suggests the activation of the inflammasomes by the venom of *Bitis arietans* and by Kn-Ba, which will be investigated in more detail in future studies.

## 1. Introduction

The global burden of snake envenoming is estimated at around 400,000 cases/year, with 20,000 deaths/year. Due to poor notifications, 1,800,000 envenomations/year and 94,000 deaths/year may occur [1]. It is estimated that, on the African continent, more than 60% of these accidents happen in sub-Saharan Africa, of which 95% occur in rural regions, leading to about 12,000 to 32,000 deaths and more than 9000 amputations related to post-envenoming complications [1,2].

Except in some islands, at high altitudes, and in regions with long-lasting snow seasons, venomous snake species are distributed worldwide, living naturally in intimate contact with human populations [3]. In the different global regions, the number of venomous snake species is large and varied. In the Middle East and North Africa, 17 snake species are found, and in sub-Saharan Africa, encompassing the Central, East, South, and West regions of the African continent, 26 species are found. Among these snakes, the *Bitis* genus includes six species, which, due to the number and severity of envenomation incidents, are important: *B. arietans*, *B. somalica*, *B. parviocula*, *B. gabonica*, *B. rhinoceros,* and *B. nasicornis* [4,5,6]. *B. arietans* is found in North Africa and in all sub-Saharan regions and is considered one of the most relevant snakes of medical importance in the African continent [4,7,8,9].

Local and systemic symptoms are observed in human victims of *Bitis arietans* bite. Intense pain, blistering, edema, ecchymosis, hemorrhage, draining lymph node hypertrophy, and necrosis are the common local symptoms. The systemic symptoms are fever, neutrophilic leukocytosis, thrombocytopenia, hemolysis, and bleeding. These systemic symptoms result in anemia, lower resistance to infections, diffuse hemorrhages, myocardial injury, coagulopathy, blood hypotension, and sometimes permanent injury of the affected body regions. All of these can cause death [9,10,11,12,13].

The *Bitis arietans* venom (BaV) is a complex mixture of proteins (±90–95%), peptides, carbohydrates, nucleic-acid-derived segments, metallic cations, biogenic amines, lipids, and free amino acids. Among the proteins, there are substantial quantities of enzymes, such as snake venom serine proteases (SVSPs), snake venom metalloproteases (SVMPs), and phospholipase-A_2_. Aside from some non-enzymatic proteins such as disintegrins, type-C lectins, cystatins, and type-Kunitz protease inhibitors are also found [14,15,16,17]. More recently, our group showed the presence of some new and already known proline-rich peptides in BaV, also known as bradykinin-potentiating peptides (BPPs) [18].

Among the most common components of BaV are proteases, specially SVSPs and SVMPs, which represent 58% of the venom composition [15,16,17]. These two protease classes, already studied in other snake venoms, such as *Bothrops atrox* [19,20,21] and *Bothrops jararaca* [22,23,24], have been identified as important toxins for the local and systemic effects observed in envenomation.

Due to the importance of these molecules, our group has been working on the purification and characterization of SVSPs and SVMPs present in the BaV. Thus, initially, a serine protease with fibrinogenolytic and kinin-releasing activities, named Kn-Ba, was purified and characterized in vitro. Kn-Ba is also recognized by antibodies (Abs) present in the horse-serum anti-*Bitis* spp. venoms [25].

Based on the importance of the inflammatory process in snake envenomation [11,23,26,27,28,29], the in vivo inflammation induced by BaV was studied in mice peritoneal cavities. BaV induced local tissue vessel dilatation followed by plasma infiltration; erythrocytes and neutrophils accumulation with simultaneous production of the eicosanoids LTB_4_, LTC_4_, TXB_2_, and PGE_2_; as well as the local and systemic production of IL-6 and MCP-1. In general, this study demonstrated that the BaV contains toxins that trigger the inflammatory process, which is partially dependent on lipid mediators [30].

In this context, aiming to better understand the inflammatory mechanisms involved in *Bitis arietans* envenomation, the present study focused on the identification of inflammatory mediators induced by BaV and Kn-Ba in human macrophages differentiated from THP-1 cells (THP-1 macrophages).

## 2. Results

### 2.1. THP-1 Macrophage Differentiation

Human non-adherent pre-monocytes of the THP-1 lineage were cultured in supplemented RPMI medium at a density of 2–8 × 10^5^ cells/mL (Supplementary Figure S1A). In these conditions, the cells grew in clumps and showed a high rate of proliferation, doubling the number of cells within 2–3 days. When the cells reached high concentrations, not exceeding 1 × 10^6^ cells/mL, live cells were differentiated in macrophages using PMA (Supplementary Figure S1B). In contrast to monocytes, differentiated macrophages become adherent and relatively larger than undifferentiated cells, stopping proliferation and spreading.

To confirm the differentiation, the expression of CD11b was evaluated by flow cytometry. CD11b, which together with CD18 forms the inactive C3b (iC3b) receptor, known as CR3 receptor, has been reported to be induced during differentiation of monocytes into macrophages. CR3 has important functions both as an adhesion molecule and a membrane receptor mediating recognition of different ligands [31,32,33]. As showed in Supplementary Figure S1C, only differentiated macrophages expressed CD11b, confirming the differentiation of these cells for further treatments.

### 2.2. Total Protein and Endotoxin Contents Determination in BaV and Kn-Ba

For THP-1 macrophage treatments, stimuli stock solutions were prepared at a concentration of 5 mg/mL (BaV) and 26 µg/mL (Kn-Ba) in sterile PBS, pH 7.2. The determined endotoxin concentrations were <0.1 UE/µg in both samples, which can be considered as an acceptable endotoxin level [34].

### 2.3. Effects of BaV and Kn-Ba on THP-1 Macrophage Viabilities

To evaluate the cytotoxic effects caused by treatments with BaV and Kn-Ba, the release of lactate dehydrogenase (LDH) in culture supernatants was evaluated. After the incubation with 0.5–15 µg of BaV and 0.5–1 µg of Kn-Ba for 24 h, 48 h, and 72 h, cell viability was greater than 90% (Supplementary Figure S2).

### 2.4. BaV Induces the Production of TNF and IL-1β in THP-1 Macrophages

**Figure 1:**
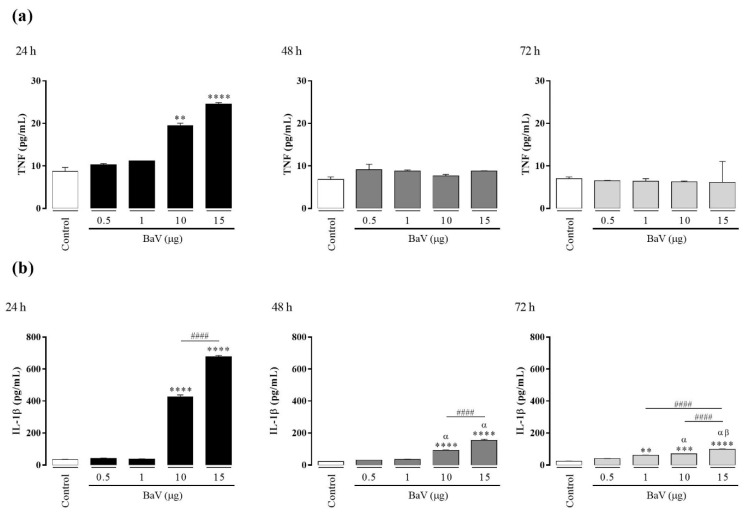
Cytokine production in THP-1 macrophages induced by BaV. THP-1 macrophages (2 × 10^5^ cells/well) were treated with different concentrations of BaV (0.5 to 15 µg/well) to induce cytokine production. Cells incubated only with culture medium were used as control. After 24 h, 48 h, and 72 h, the cytokine production was evaluated in culture supernatant by CBA. (**a**) TNF and (**b**) IL-1β. Results expressed as mean of duplicates ± SEM and analyzed by one-way ANOVA followed by Tukey’s post-test (each period compared with respective control) or two-way ANOVA followed by Tukey’s post-test (comparison between different periods of treatment). (*) Significant difference in relation to the respective control. (#) Significant difference between concentrations of BaV on each period. Symbols indicate differences between periods of treatment: (α) difference of 48 h or 72 h compared to 24 h; (β) difference from 72 h to 48 h. *p* < 0.01 (**); *p* < 0.001 (***); and *p* < 0.0001 (**** and ^####^).

The concentration of cytokines IL-1β, IL-6, IL-10, IL-12, and TNF were evaluated on cell-free supernatants by CBA (Cytometric Bead Array) after 24 h, 48 h, and 72 h of treatments with BaV. As shown in Figure 1, BaV induced the production of TNF and IL-1β in a concentration-dependent manner. The production of TNF was detected only after 24 h in response to the two highest doses of BaV (10 µg and 15 µg/well). On the other hand, the maximum production of IL-1β, also in response to the two highest doses of BaV, was reached in 24 h, then decreased after 48 h and 72 h of treatment. BaV did not induce the production of IL-6, IL-10, or IL-12.

### 2.5. BaV Induces the Production of RANTES and IL-8 in THP-1 Macrophages

**Figure 2:**
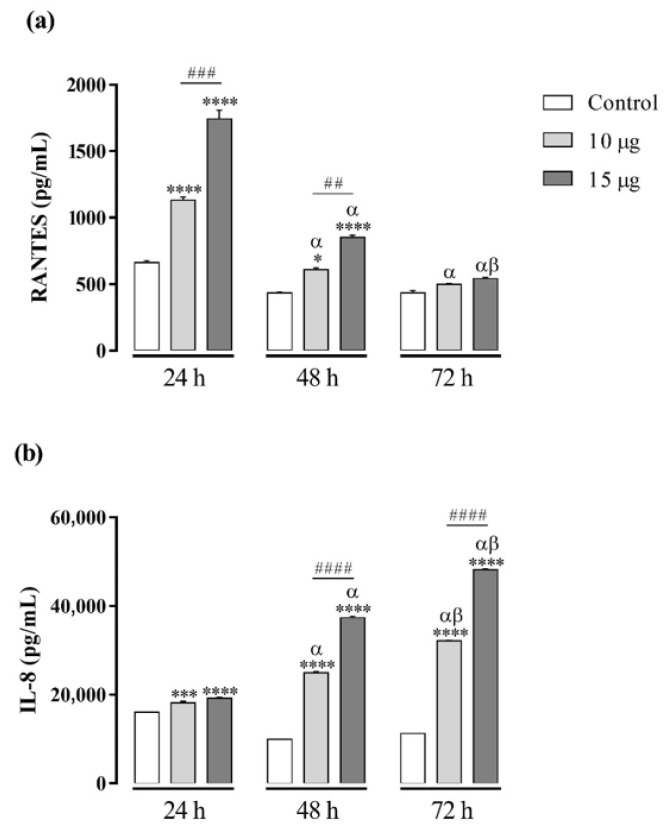
Chemokine productions in THP-1 macrophages induced by BaV. THP-1 macrophages (2 × 10^5^ cells/well) were treated with two concentrations of BaV (10 and 15 µg/well). Cells incubated only with culture medium were used as control. After 24 h, 48 h, and 72 h, the production of chemokines were evaluated in culture supernatant by CBA. (**a**) RANTES and (**b**) IL-8. Results expressed as mean of duplicates ± SEM and analyzed by one-way ANOVA followed by Tukey’s post-test (each period compared with respective control) or two-way ANOVA followed by Tukey’s post-test (comparison between different periods of treatment). (*) Significant difference in relation to the respective control. (#) Significant difference between concentrations of BaV on each period. Symbols indicate differences between periods of treatment: (α) difference of 48 h or 72 h compared to 24 h; (β) difference from 72 h to 48 h. *p* < 0.05 (*); *p* < 0.01 (^##^); *p* < 0.001 (*** and ^###^); and *p* < 0.0001 (**** and ^####^).

As the two highest doses of BaV (10 and 15 µg/well) were able to induce the cytokines production without affecting the cell viability, these doses were selected to evaluate the production of chemokines CXCL8/IL-8, CCL5/RANTES, CXCL9/MIG, CCL2/MCP-1, and CXCL10/IP-10 in cell-free supernatants by CBA after 24 h, 48 h, and 72 h of treatments with BaV. BaV induced the production of RANTES and IL-8. RANTES was produced in a dose-dependent manner after 24 h, decaying thereafter. IL-8, in contrast, started to be produced—also in a dose-dependent manner—mainly after 48 h and continuing after 72 h (Figure 2). BaV did not induce the production of CXCL9/MIG, CCL2/MCP-1, and CXCL10/IP-10.

### 2.6. Kn-Ba Induces the Production of TNF, IL-6, and IL-1β in THP-1 Macrophages

**Figure 3:**
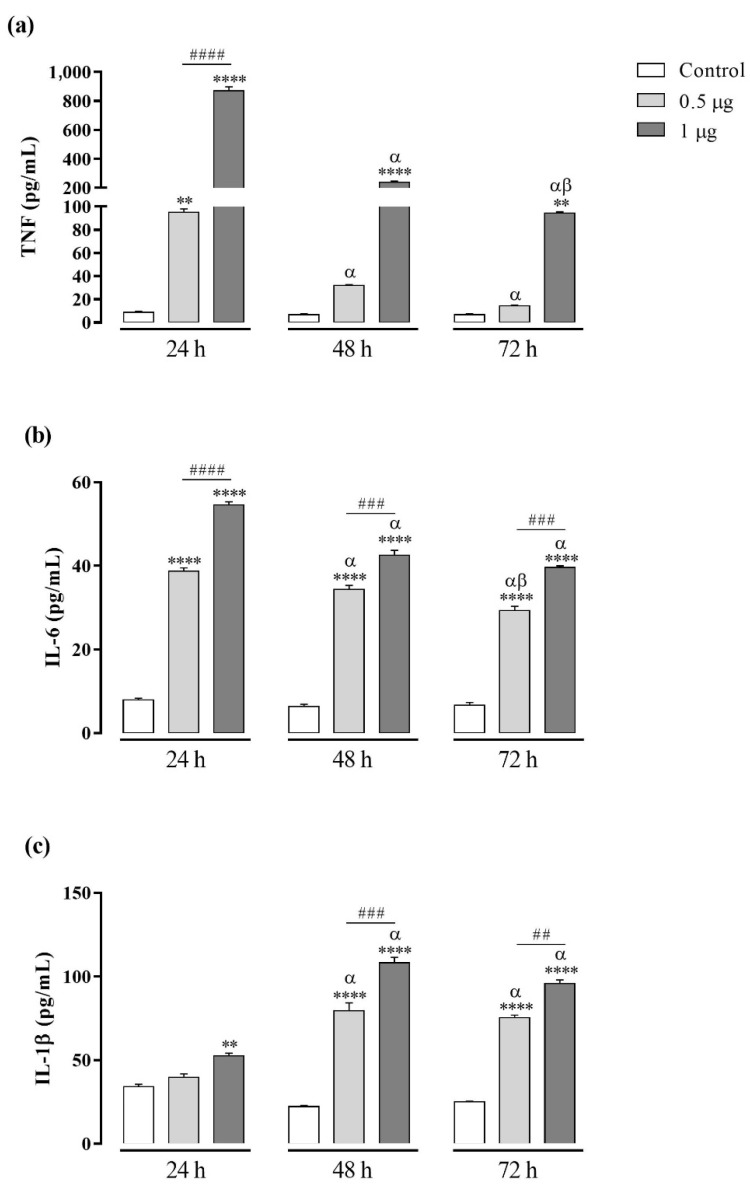
Cytokine production in THP-1 macrophages induced by Kn-Ba. THP-1 macrophages (2 × 10^5^ cells/well) were treated with two concentrations of Kn-Ba (0.5 and 1 µg/well). Cells incubated only with culture medium were used as control. After 24 h, 48 h, and 72 h the production of cytokines were evaluated in culture supernatant by CBA. (**a**) TNF, (**b**) IL-6 and (**c**) IL-1β. Results expressed as mean of duplicates ± SEM and analyzed by one-way ANOVA followed by Tukey’s post-test (each period compared with respective control) or two-way ANOVA followed by Tukey’s post-test (comparison between different periods of treatment). (*) Significant difference in relation to the respective control. (#) Significant difference between concentrations of Kn-Ba on each period. Symbols indicate differences between periods of treatment: (α) difference of 48 h or 72 h compared to 24 h; (β) difference from 72 h to 48 h. *p* < 0.01 (** and ^##^); *p* < 0.001 (^###^); and *p* < 0.0001 (**** and ^####^).

The concentrations of cytokines IL-1β, IL-6, IL-10, IL-12, and TNF were evaluated on cell-free supernatants by CBA after 24 h, 48 h, and 72 h of treatments with Kn-Ba. Kn-Ba induced the production of TNF and IL-1β, and, unlike BaV, it was also able to induce IL-6 production. At the two doses used (0.5 and 1 µg), Kn-Ba induced the production of these chemokines in concentration-dependent manners. The TNF and IL-6 production were detected after 24 h, decreasing thereafter, whereas the production of IL-1 β was accentuated after 48 h and maintained after 72 h (Figure 3). Kn-Ba did not induce the production of IL-10 or IL-12.

### 2.7. Kn-Ba Induces the Production of IP-10, MCP-1, RANTES, and IL-8 in THP-1 Macrophages

**Figure 4:**
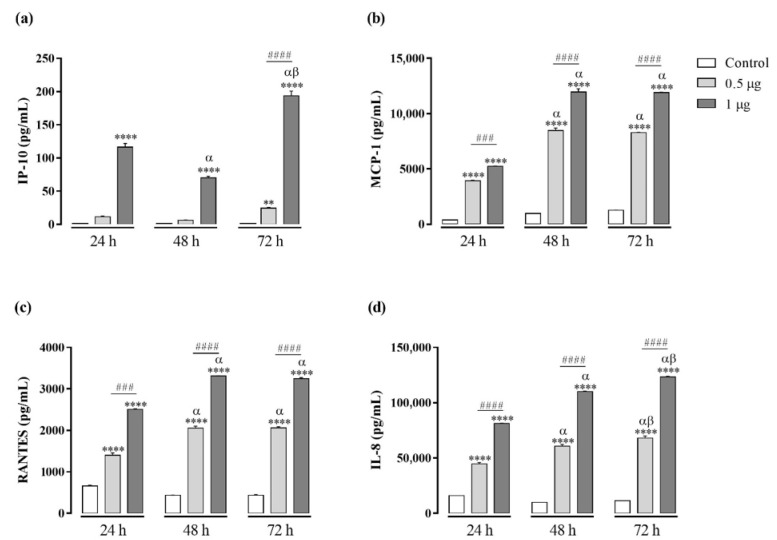
Chemokine productions in THP-1 macrophages induced by Kn-Ba. THP-1 macrophages (2 × 10^5^ cells/well) were treated with two concentrations of Kn-Ba (0.5 and 1 µg/well). Cells incubated only with culture medium were used as control. After 24 h, 48 h, and 72 h, the production of chemokines were evaluated in culture supernatant by CBA. (**a**) IP-10, (**b**) MCP-1, (**c**) RANTES and (**d**) IL-8. Results expressed as mean of duplicates ± SEM and analyzed by one-way ANOVA followed by Tukey’s post-test (each period compared with respective control) or two-way ANOVA followed by Tukey’s post-test (comparison between different periods of treatment). (*) Significant difference in relation to the respective control. (#) Significant difference between concentrations of Kn-Ba on each period. Symbols indicate differences between periods of treatment: (α) difference of 48 h or 72 h compared to 24 h; (β) difference from 72 h to 48 h. *p* < 0.01 (**); *p* < 0.001 (^###^); and *p* < 0.0001 (**** and ^####^).

The concentrations of chemokines CXCL8/IL-8, CCL5/RANTES, CXCL9/MIG, CCL2/MCP-1, and CXCL10/IP-10 were evaluated in cell-free supernatants by CBA after 24 h, 48 h, and 72 h of treatments with Kn-Ba (0.5 and 1 µg). Just like BaV, Kn-Ba induced the production of RANTES and IL-8, but was also able to induce the production of IP-10 and MCP-1, in time- and concentration-dependent manners (Figure 4).

### 2.8. Kn-Ba Induced High Levels of TNF and IL-6, Whereas BaV Is Involved in the IL-1β Production in THP-1 Macrophages

**Figure 5:**
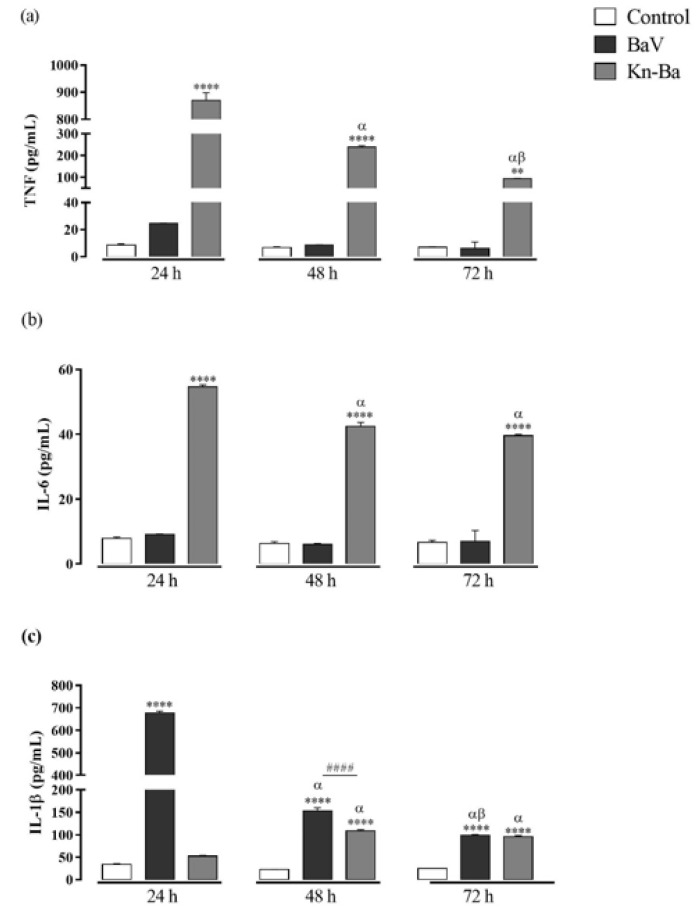
Comparison between cytokine productions induced by higher doses of BaV and Kn-Ba. THP-1 macrophages (2 × 10^5^ cells/well) were treated with higher concentrations of BaV (15 µg/well) and Kn-Ba (1 µg/well). Cells incubated only with culture medium were used as control. After 24 h, 48 h, and 72 h, the production of cytokines was evaluated in culture supernatant by CBA. (**a**) TNF, (**b**) IL-6 and (**c**) IL-1β. Results expressed as mean of duplicates ± SEM and analyzed by one-way ANOVA followed by Tukey’s post-test (each period compared with respective control) or two-way ANOVA followed by Tukey’s post-test (comparison between different periods of treatment). (*) Significant difference in relation to the respective control. (#) Significant difference between stimuli on each period. Symbols indicate differences between periods of treatment: (α) difference of 48 h or 72 h compared to 24 h; (β) difference from 72 h to 48 h. *p* < 0.01 (**); and *p* < 0.0001 (**** and ^####^).

The cytokine concentrations produced in response to highest doses of BaV (15 µg/well) and Kn-Ba (1 µg/well) were compared. As shown in Figure 5, the increased productions of TNF and IL-6 in response to Kn-Ba were evident. However, the highest concentration of IL-1β was detected mainly in response to BaV, suggesting that this important inflammatory cytokine is produced in response to other components present in BaV.

### 2.9. Kn-Ba Induced the Production of Highest Levels of all Evaluated Chemokines in THP-1 Macrophages

**Figure 6:**
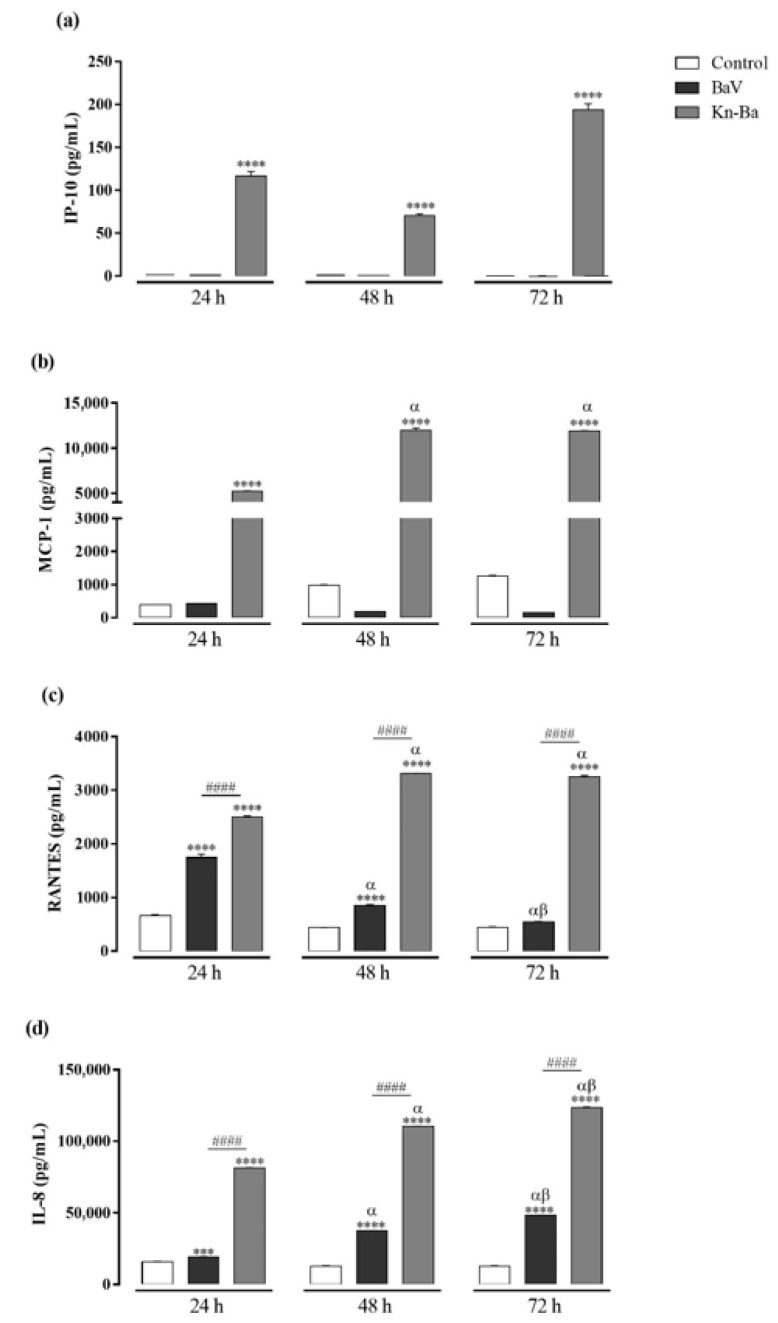
Comparison between chemokine productions induced by higher doses of BaV and Kn-Ba. THP-1 macrophages (2 × 10^5^ cells/well) were treated with higher concentrations of BaV (15 µg/well) and Kn-Ba (1 µg/well). Cells incubated only with culture medium were used as control. After 24 h, 48 h, and 72 h, the production of chemokines was evaluated in culture supernatant by CBA. (**a**) IP-10, (**b**) MCP-1, (**c**) RANTES and (**d**) IL-8. Results expressed as mean of duplicates ± SEM and analyzed by one-way ANOVA followed by Tukey’s post-test (each period compared with respective control) or two-way ANOVA followed by Tukey’s post-test (comparison between different periods of treatment). (*) Significant difference in relation to the respective control. (#) Significant difference between stimuli on each period. Symbols indicate differences between periods of treatment: (α) difference of 48 h or 72 h compared to 24 h; (β) difference from 72 h to 48 h. *p* < 0.001 (***); and *p* < 0.0001 (**** and ^####^).

The chemokine concentrations produced in response to the highest doses of BaV (15 µg/well) and Kn-Ba (1 µg/well) were compared. As depicted in Figure 6, the higher productions of the chemokines IP-10, MCP-1, RANTES, and IL-8 were detected in response to Kn-Ba.

### 2.10. BaV, But Not Kn-Ba, Induced the Production of PGE_2_

**Figure 7:**
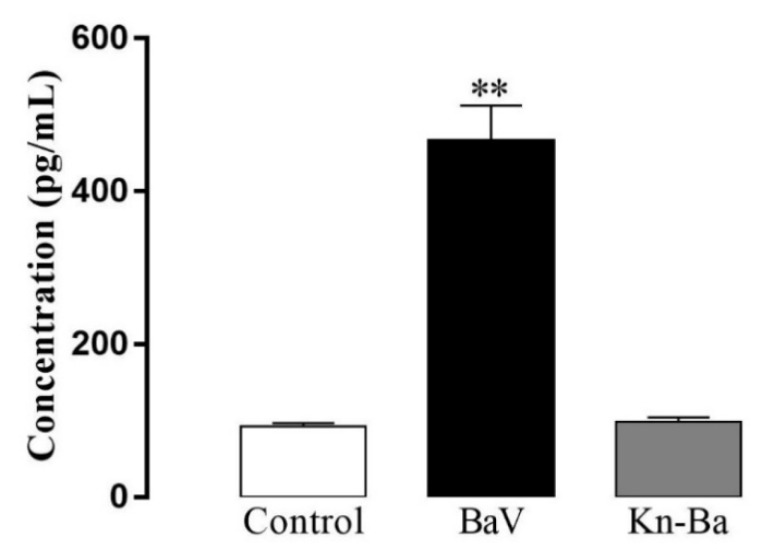
Production of PGE_2_. THP-1 macrophages (2 × 10^5^ cells/well) were treated with higher concentrations of BaV (15 µg/well) and Kn-Ba (1 µg/well). Cells incubated only with culture medium were used as control. After 30 min, the production of PGE_2_ was evaluated by ELISA. Results expressed as mean of duplicates ± SEM and analyzed by one-way ANOVA followed by Tukey’s post-test. (Asterisk) Significant difference from control and Kn-Ba. *p* < 0.01 (**).

The concentrations of the eicosanoids PGE_2_ and LTB_4_ were evaluated in cell-free supernatants by competitive ELISA after 30 min of treatments with higher doses of BaV (15 µg/well) and Kn-Ba (1 µg/well). BaV, but not Kn-Ba, induced the production of PGE_2_ (Figure 7). No stimulus was able to induce the production of LTB_4_ (data not shown).

## 3. Discussion

Inflammation is the hallmark of envenomation by snakes in the Viperidae family. The activation of the inflammatory process and its cascade of events play an important role in the pathogenesis of envenomation, in the clinical picture, and in the outcome of the accident. In this sense, the envenomation by *Bothrops asper* [35,36], *B. atrox* [37,38], and *B. jararaca* [39,40], which, among other characteristic changes such as hemostatic and cardiovascular disorders, induce a prominent inflammatory response, which has been associated not only with local damage but also with systemic disturbances caused by Viperidae venoms [11,29]. Therefore, given the importance in understanding the inflammatory response induced by the BaV as a tool for the development of complementary therapies, or the improvement of *B. arietans* antivenoms, we show that this venom was capable of inducing, in vitro, the production of several inflammatory mediators, including cytokines, chemokines, and lipid mediators.

Our previous in vivo results demonstrated that BaV induces inflammation with the participation of diverse endogenous inflammatory mediators produced by residents or derivatives of recently migrated blood cells [30]. Among these cells, macrophages became candidates for target cells in the present study of cell inflammation induced by BaV and Kn-Ba, since macrophages play a key role in inflammation [41] and also participate in the inflammatory response related to other snake envenomations [22,23,42]. In this sense, studies have shown that in the absence of macrophages, the recruitment of immune cells to the site of inflammation, especially neutrophils, is impaired [22,23].

Macrophages express, on their cell surface, receptors named pattern-recognizing receptors (PRRs), which recognize the molecular domains such as pathogen-associated molecular patterns (PAMPs) expressed by pathogens, virus, fungus, bacteria; and damage-associated molecular patterns (DAMPs), which are expressed by host-damaged molecules [43,44,45]. In addition, the PRRs can also recognize venom-associated molecular patterns, also known as VAMPs. The term VAMP was coined by Brazilian researchers, who showed that mice deficient in TLR (Toll-like receptor)-2 and TLR-4, two important PRRs, as well as the adapter molecule CD14, produce fewer inflammatory mediators in response to the *Tityus serrulatus* scorpion venom, including decreased levels of IL-6 and TNF-α [46].

In view of the important role of macrophages in the inflammatory process, we used THP-1 macrophages to evaluate the in vitro pro-inflammatory properties of BaV and Kn-Ba. To support the homogeneity of the experiments, the human monocytes of the THP-1 lineage were differentiated in vitro into macrophages with PMA, as described in the literature [33,47]. There are different protocols using PMA for the differentiation of THP-1 monocytes to macrophages, but in some cases the differentiation profile may be not comparable to primary monocyte-derived macrophages [48]. To overcome this inconvenience, macrophages treated with PMA were kept at rest for 4 days in culture media without PMA, which was shown to be a good alternative to induce a differentiation pattern similar to primary monocyte-derived macrophages [33]. This differentiation was evaluated by morphological analyzes such as adhesion to the support surface, spreading and emission of pseudopods, and loss of proliferation. In addition to the morphological alterations, the differentiated macrophages, but no undifferentiated THP-1 monocytes, expressed CD11b, an important surface marker of monocyte-to-macrophage maturation [31,32,33]. CD11b forms the molecular complex CD11b-CD18, known as CR3 receptor, which is expressed in macrophages and in other leucocytes and binds to several ligands, such as iC3b adhered to pathogens. These promote phagocytosis [49] and expanding the macrophages’ biological activities [33]. After differentiation, the THP-1 macrophages were used as target cells in the studies with BaV and its purified serine protease, Kn-Ba.

In sequence, the effects of BaV and Kn-Ba as inflammation-inducers were demonstrated by the production of the cytokines TNF, IL-6, IL-1β; and the chemokines IP-10, MCP-1, RANTES, and IL-8; besides the lipid mediator PGE_2_.

The role of purified SVSP on the inflammation induced by snake venoms has been extensively studied [50,51,52]. However, although SVSPs presents a high degree of similarity between their amino acids sequences, their functions may differ [53], so that not all are involved with the inflammatory response. In this sense, two SVSPs purified from *Bothrops pirajai* venom, named BpirSP27 and BpirSP41, seem to be not involved in the inflammatory events related to *B. pirajai* envenomation, such as edema, pain, and leukocyte recruitment [54]. In contrast, it was showed that SVSPs from the venoms of *Bothrops alternatus* and *B. moojeni* are able to promote edema and pain, two classic signs of inflammation [50]. SVSPs from *Bothrops asper* can also activate endogenous matrix metalloproteases [51], while SVSPs from *Crotalus durissus terrificus* venom induced edema and the increased expression of COX-2 and PGE_2_ production [52]. In agreement with these finds, here we show that Kn-Ba induces the production of inflammatory cytokines and chemokines by THP-1-derived human macrophages, indicating its participation in BaV-induced inflammation.

Our results show differences between the type and concentration of some inflammatory mediators produced in response to treatments with BaV or purified Kn-Ba. In this sense, it can be suggested that the mediators produced only after stimuli with BaV, but not with Kn-Ba, as in the case of PGE_2_, were generated by other components present in the venom. However, here we also show that some mediators, more precisely IL-6, IP-10, and MCP-1, were produced only after stimulation with Kn-Ba. This is a complex issue involving both the composition of the venom itself and the immune response triggered against the components of the whole venom. It is well known that BaV is a complex mixture of proteins (±90–95%), which includes, besides enzymes such as SVSP and SVMPs, non-enzymatic proteins as Kunitz-type proteases inhibitors [14,15,16,17]. Therefore, it can be suggested that the activity of some proteases could be hindered by endogenous inhibitors present in the venom itself. Thus, it is possible that purified Kn-Ba inducing the production of different inflammatory mediators, in a greater degree than BaV, may be due to the absence of these inhibitors. In addition, the different components of BaV can act in a different way in immune cells, making the inflammatory response diverse from the one developed only against purified Kn-Ba.

Among the inflammatory mediators produced in response to BaV and Kn-Ba, the cytokine IL-1β is noteworthy. According to literature data, after the activation of macrophages via TLRs, a cascade of intracellular signaling begins. This cascade may culminate in the activation of an important transcription factor named NF-κB, which is responsible for the expression of diverse inflammatory genes, including TNF-α, IL-6, iNOS, and also pro IL-1β [45,55,56]. Pro IL-1β is a zymogen whose activation and later secretion depends on the inflammasomes’ activation, a multiprotein complex formed due to cell activation by the recognition of patterns via cytosolic PRRs of the NLR family (NOD-like receptors) [57,58]. An important subfamily of NLRs, called NALP, is involved in the induction of the inflammatory response via cytokines of the IL-1 family, including IL-1β, IL-8, and IL-33. Recognition via NALP promotes the formation of inflammasomes, which are responsible for the activation of inflammatory caspases 1, 4, and 5 in humans, and caspases-1, 11, and 12 in mice, which can convert pro-IL-1β into mature IL-1β [59,60]. IL-1β is a highly inflammatory cytokine produced during various inflammatory conditions, which mediates innate and adaptative immune responses by promoting acute phase response and recruiting inflammatory cells [61,62]. The overproduction of IL-1β is harmful and can trigger autoimmune diseases [63,64].

Here, we show that both BaV and Kn-Ba induced the production and secretion of IL-1β in the supernatant of macrophage cultures, suggesting the participation of inflammasomes in the inflammatory process related to the *Bitis arietans* envenomation. In addition, as higher concentrations of this cytokine were detected after treatment with BaV, our results indicate there are other components present in BaV, besides Kn-Ba, which may be involved in the possible inflammasome activation.

IL-1β can act synergistically with other cytokines produced by human macrophages to amplify *Bitis-arietans*-related inflammations, such as with TNF-α, activating the endothelium and inducing vasodilation and increased vascular permeability, and, with IL-6, activating hepatocytes and inducing the production of acute phase proteins, which can activate the complement system and act as opsonins, facilitating phagocytosis by macrophages and neutrophils [45].

Summing up, our study showed that BaV and Kn-Ba are able to induce the activation and the production of inflammatory mediators in THP-1-derived human macrophages, and this is the first suggestion that the inflammasomes may play a role in *Bitis arietans* envenomation.

## 4. Conclusions

**Figure 8:**
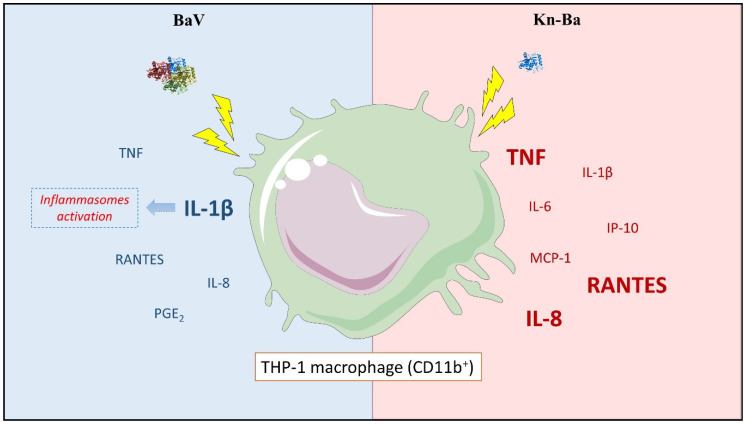
Comparative overview of the inflammatory profiles induced by BaV and Kn-Ba in THP-1 macrophages. THP-1 macrophages were treated with BaV (left; blue) and Kn-Ba (right; red) resulting in the synthesis and secretion of the indicated cytokines, chemokines, and PGE_2_. The mediators highlighted in larger size were produced by both stimuli, but at higher levels at the evidenced side. Both stimuli induced the production and release of IL-1β, indicating the inflammasomes pathway activation during BaV envenomation. However, the highest production of this cytokine in response to BaV indicated the participation of other venom components in this process. The schematic art pieces used in this figure were provided by Servier Medical art. Servier Medical Art by Servier is licensed under a Creative Commons Attribution 4.0 Unported License.

Our study showed, for the first time, the pro-inflammatory effects of BaV and Kn-Ba upon THP-1 derived human macrophages. Both stimuli are responsible for the production of the cytokines TNF and the chemokines RANTES and IL-8. However, the significant production of these mediators occurred in response to Kn-Ba. Only Kn-Ba was able to induce the production of IL-6, MCP-1, and IP-10, whereas PGE_2_ was produced only in response to BaV. Finally, although both stimuli induced the production of IL-1β, suggesting that the inflammasomes may play a role in BaV envenomation, the highest production of this cytokine in response to BaV suggests the participation of other venom components in this process (Figure 8). These results together with previous data published by our group describing the biological actions of Kn-Ba and BaV-induced inflammation in vivo help us to better understand the inflammatory mechanisms involved in this envenomation, and to list important toxins for the future development of antivenoms.

## 5. Materials and Methods

### 5.1. BaV

Lyophilized *B. arietans* venom (BaV) was purchased from Venom Supplies, Tanunda, Australia. The venoms were obtained from male and female snakes of different ages, captured in South Africa and maintained in captivity. Stock solutions were prepared in sterile phosphate-buffered saline (PBS, 8.1 mM Na_2_HPO_4_; 1.5 mM KH_2_PO_4_; 137 mM NaCl; 2.7 mM KCl, pH 7.2) and stored at −80 °C.

### 5.2. Kn-Ba

Kn-Ba was purified from BaV as previously described [25]. Purified Kn-Ba was diluted in sterile PBS pH 7.2 and stored at −80 °C.

### 5.3. Total Protein Quantification

Total protein concentration of BaV and Kn-Ba were measured by the bicinchoninic acid method [65] using the Pierce BCA Protein Assay kit (Pierce Biotechnology, Waltham, IL, USA), according to manufacture instructions. A standard curve was prepared using increased concentrations (0–2000 µg/mL) of pure bovine serum albumin (BSA, Sigma Aldrich, St. Louis, MO, USA) diluted in sterile PBS pH 7.4. Absorbances were obtained in the plate spectrophotometer (ELX 800, Biotek Instruments, Winooski, VT, USA) at ƛ540 nm.

### 5.4. Endotoxin Contents Determination

Samples of BaV (20 µg/mL) and Kn-Ba (1 µg/mL) were diluted in sterile PBS, pH 7.2, and the endotoxin presence was analyzed by the Microbiological Quality Control of Butantan Foundation using the PYROGENT TM Gel clot LAL Assays kit (Lonza, Walkersville, MD, USA), according to manufacture instructions. Endotoxin concentration was estimated by comparing with an *Escherichia coli* LPS standard (0.125 UE/mL).

### 5.5. Human THP-1 Pre-Monocyte Culture

The non-adherent human pre-monocyte of the THP-1 cell lineage was acquired from the Rio de Janeiro Cell Bank (Rio de Janeiro, RJ, Brazil, cat.: BCRJ: 0234) and cultured according to the manufacture instructions. The suspension cells were cultured in 75 cm^3^ culture flasks (Corning Inc., New York, NY, USA) with RPMI-1640 medium (Gibco, Invitrogen Corp., Waltham, MA, USA) supplemented with 23 mM NaHCO_3_, 13 mM C_6_H_12_O_6_, 10 mM Hepes, 2 mM L-glutamine, 1 mM sodium pyruvate, 10% FBS (Fetal Bovine Serum, Cultilab, São Paulo, SP, Brazil), 100 U/mL of penicillin, and 100 µg/mL of streptomycin (Gibco, Invitrogen Corp., Waltham, MA, USA). The cell was maintained at 37 °C in an atmosphere containing 5% CO_2_ at a density of 2–8 × 10^5^ cells/mL, not exceeding the concentration of 1 × 10^6^ cells/mL. Total cell number and cell viability were periodically monitored in a Neubauer chamber by Trypan blue exclusion (Trypan blue 0.4% in PBS pH 7.2; 1:1; *v*/*v*).

### 5.6. Human THP-1 Pre-Monocyte Differentiation into THP-1 Macrophages

Human pre-monocytes were differentiated into macrophages using Phorbol 12-Myristate 13-Acetate (PMA, Sigma Aldrich, St. Louis, MO, USA), according to protocol described by Daigneault and colleagues (2010) [33]. Briefly, live cells were transferred to 24 culture wells plates containing 1 mL/well of supplemented RPMI medium containing PMA (100 ng/mL) at a density of 2 × 10^5^ cells/well. The cell was incubated at 37 °C in an atmosphere containing 5% CO_2_ during 72 h. After the fourth day, PMA-containing medium was replaced by medium without PMA (2 mL/well), and the cells were kept at rest for 4 more days. At the end of the rest period, macrophage adhesion was visualized under a phase contrast microscope (Leica DM2500, Wetzlar, Germany) and photographed.

### 5.7. CD11b Expression in Differentiated THP-1 Macrophages

The CD11b expression by differentiated THP-1 macrophages was evaluated by flow cytometry, according to the literature [32], with some modifications, as described below. THP-1 monocytes were cultured in 75 cm^3^ culture flasks until reaching a density of 8 × 10^5^ cells/mL, according to what is described in 5.5. At this time, the culture medium was replaced with medium containing 100 ng/mL of PMA, and differentiation was conducted as described in 5.6. The adherent macrophages were carefully detached with cell scraper and centrifuged (260× *g*) for 10 min at 10 °C. Contents of viable cells were determined by Trypan blue exclusion, and the cell pellet was resuspended in FACS buffer (PBS with 1% BSA and 0.01% sodium azide) at the density of 20 × 10^6^ cells/mL. Cells were transferred to 96 round-well-bottom microplates (50 µL/well) and were incubated protected from light for 30 min at 4 °C with anti-CD11b PE-labelled antibody (IgG1κ PE, Clone VIM 12, BD, Becton Dickinson, San Jose, CA, USA) or with isotypic control (IgG1κ PE, BD) diluted at 1:5 (*v*/*v*) in FACS buffer. After incubation, the cell suspensions were centrifuged (305× *g*) for 5 min at 4 °C, washed three times with FACS buffer and resuspended in 300 µL/well of FACS buffer with 1% paraformaldehyde. The data acquisition was performed in flow cytometer (FACS Aria III, Becton Dickinson, San Jose, CA, USA). In parallel, the CD11b expression was also evaluated in THP-1 monocytes as control.

### 5.8. Incubation of THP-1 Macrophages with BaV and Kn-Ba

THP-1 differentiated in 24-well plates, according to what is described in 5.4, were treated with BaV (0.5 µg, 1 µg, 10 µg, and 15 µg/well) or with Kn-Ba (0.5 µg and 1 µg/well) diluted in 500 µL/well of supplemented RPMI medium without FBS and incubated at 37 °C in an atmosphere containing 5% CO_2_ during 24 h, 48 h, and 72 h [66]. The supernatants were collected, centrifuged (405× *g*) for 5 min at 4 ºC for removal of cell debris, aliquoted, and stored at −80 °C.

### 5.9. Release of Lactate Dehydrogenase (LDH)

The cytotoxic effects after BaV and Kn-Ba treatments were evaluated by the quantification of lactate dehydrogenase (LDH) enzyme in cell-free supernatants using the kit CytoTox 96^®^ Non-Radioactive Cytotoxicity Assay (Promega, Madison, WI, USA) [67], according to the manufacturer’s instructions. Cell-free supernatants of THP-1 macrophages treated during 45 min with lysis buffer were used as a positive control of LDH release, and cells treated only with culture medium were used as LDH release background.

### 5.10. Quantification of Cytokines and Chemokines Produced by THP-1 Macrophages

The cytokines and chemokines produced by THP-1 macrophages in response to the treatments with BaV and Kn-Ba were evaluated in cell-free supernatants. For quantification of inflammatory cytokines IL-1β, IL-6, IL-10, IL-12, and TNF-α, the CBA Human Inflammatory Cytokines kit was used (BD, Bioscience, Franklin Lakes, NJ, USA), and for quantification of the chemokines CXCL8/IL-8, CCL5/RANTES, CXCL9/MIG, CCL2/MCP-1, and CXCL10/IP-10, the CBA Human Chemokines kit was used (BD, Bioscience, Franklin Lakes, NJ, USA), according to the manufacturer’s instructions. The data acquisition was performed in a flow cytometer (FACS Aria III, Becton Dickinson, San Jose, CA, USA) and analyzed by the software FACS Diva, version 6.1.3.

### 5.11. Quantification of Lipid Inflammatory Mediators Produced by THP-1 Macrophages

The concentration of the lipid mediators LTB_4_ and PGE_2_ in cell-free supernatants was quantified by competitive ELISA using specific kits (EIA, Cayman Chemical, Ann Arbor, MI, USA), according to the manufacturer’s instructions. The absorbances were determined in a spectrophotometer at λ 405/420 nm (VersaMax, Molecular Devices, San Jose, CA, USA). The eicosanoids concentrations were calculated by the interpolation on the kits’ standard curve.

### 5.12. Statistical Analysis

Data are presented as means ± standard error (SEM) and analyzed by Graph Pad Prism, version 6.0 for Windows (Graph Pad Software, San Diego, CA, USA). One-way ANOVA test and multiple comparisons by Tukey HSD were used for comparisons of one variable in more than two groups. For comparisons of two or more variables, two-way ANOVA followed by Tukey HSD were used. For all tests, the values *p* < 0.05 were considered significant. The assays were conducted in duplicate and repeated at least twice in independent days.

## Supplementary Materials

The following are available online at https://www.mdpi.com/article/10.3390/toxins13120906/s1, Figure S1. Differentiation of THP-1 macrophages. (A) Human pre-monocytes of the THP-1 lineage were cultured in 75 cm^3^ bottles containing supplemented RPMI medium. The cells were kept in an incubator at 37 °C and 5% CO_2_, at a concentration of 2–8 × 10^5^ cells/mL. (B) Macrophages were differentiated from THP-1 pre-monocytes with 100 ng/mL PMA for 3 days, followed by a 4-day rest period in the absence of PMA. 200× magnification. (C) CD11b expression in differentiated THP-1 macrophages. Adherent THP-1 macrophages differentiated with PMA and pre-monocytes in suspension were analyzed for CD11b expression by flow cytometry. Result expressed as mean of duplicates ± SEM and analyzed by two-way ANOVA followed by Tukey’s post-test. (*) Significant difference in relation to the respective controls (*p* < 0.0001). Figure S2. Cell cytotoxicity. Differentiated THP-1 macrophages was treated with 0.5–15 µg/well of BaV or with 0.5 and 1 µg/well Kn-Ba for 24 h, 48 h, and 72 h, and cell viability was evaluated by the quantification of lactate dehydrogenase (LDH) enzyme in cell-free supernatants. Results expressed as mean of duplicates ± SEM and analyzed by one-way ANOVA followed by Tukey’s post-test. (*) Significant difference in relation to the respective control. *p* < 0.01 (**), and *p* < 0.0001 (****).
