# Supplementary material for: Bitis arietans Snake Venom and Kn-Ba, a Snake Venom Serine Protease, Induce the Production of Inflammatory Mediators in THP-1 Macrophages
Source: Toxins (Basel). 2021 Dec 16;13(12):906. doi: 10.3390/toxins13120906 (PMC8705107; doi:10.3390/toxins13120906)
Supplement: Supplementary file 1 [file toxins-13-00906-s001.zip › Supplementary Figure S1.pdf]

Toxins **2021**, *13*, 906.

Bitis arietans Snake Venom and Kn-Ba, a Snake Venom Serine Protease, Induce the Production of Inflammatory Mediators in THP-1 Macrophages

Ângela Alice Amadeu Megale [1*], Fabio Carlos Magnoli [1], Felipe Raimondi Guidolin [1], Kemily Stephanie Godoi [1], Fernanda Calheta Vieira Portaro [2*] and Wilmar Dias-da-Silva [1*]

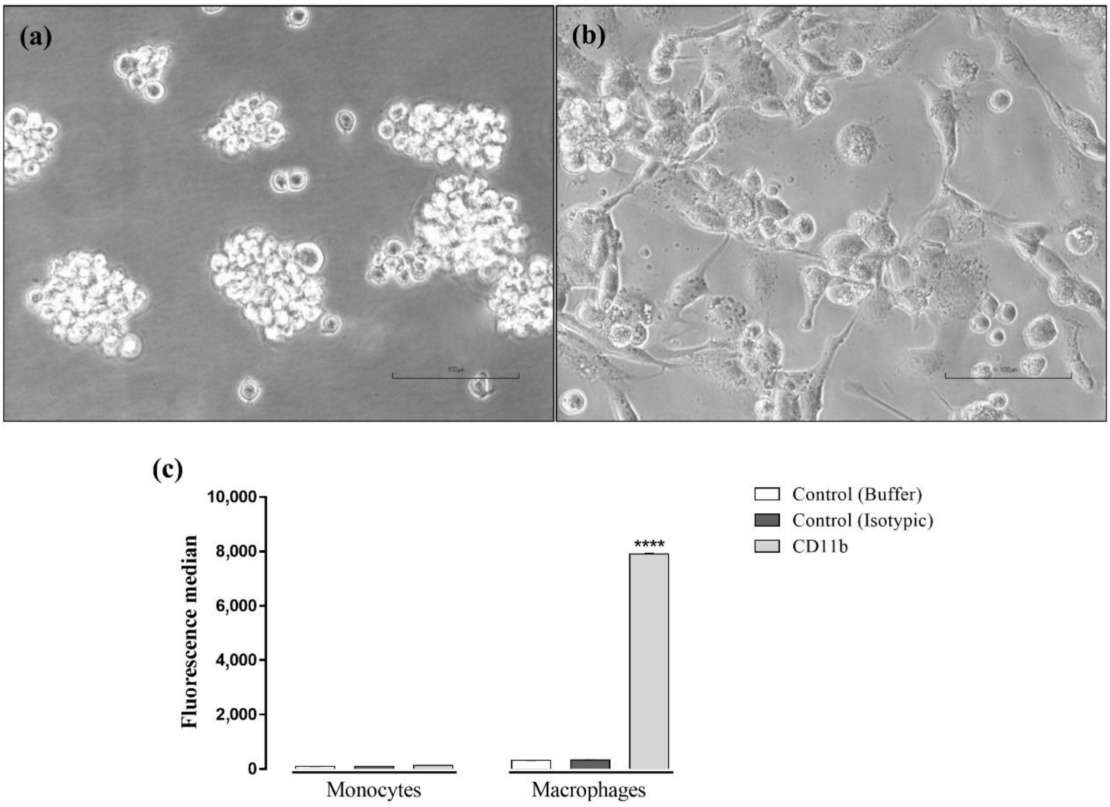

Figure S1. Differentiation of THP-1 macrophages. (A) Human pre-monocytes of the THP-1 lineage were cultured in 75 cm³ bottles containing supplemented RPMI medium. The cells were kept in an incubator at 37 °C and 5% CO_2, at a concentration of 2–8 × 10^5 cells/mL. (B) Macrophages were differentiated from THP-1 pre-monocytes with 100 ng/mL PMA for 3 days, followed by a 4-day rest period in the absence of PMA. 200× magnification. (C) CD11b expression in differentiated THP-1 macrophages. Adherent THP-1 macrophages differentiated with PMA and pre-monocytes in suspension were analyzed for CD11b expression by flow cytometry. Result expressed as mean of duplicates ± SEM and analyzed by two-way ANOVA followed by Tukey's post-test. (*) Significant difference in relation to the respective controls ($p < 0.0001$).
