# Supplementary material for: Bitis arietans Snake Venom and Kn-Ba, a Snake Venom Serine Protease, Induce the Production of Inflammatory Mediators in THP-1 Macrophages
Source: Toxins (Basel). 2021 Dec 16;13(12):906. doi: 10.3390/toxins13120906 (PMC8705107; doi:10.3390/toxins13120906)
Supplement: Supplementary file 1 [file toxins-13-00906-s001.zip › Supplementary Figure S2.pdf]

Toxins **2021**, *13*, 906.

Bitis arietans Snake Venom and Kn-Ba, a Snake Venom Serine Protease, Induce the Production of Inflammatory Mediators in THP-1 Macrophages

Ângela Alice Amadeu Megale [1]*, Fabio Carlos Magnoli [1], Felipe Raimondi Guidolin [1], Kemily Stephanie Godoi [1], Fernanda Calheta Vieira Portaro [2]* and Wilmar Dias-da-Silva [1]*

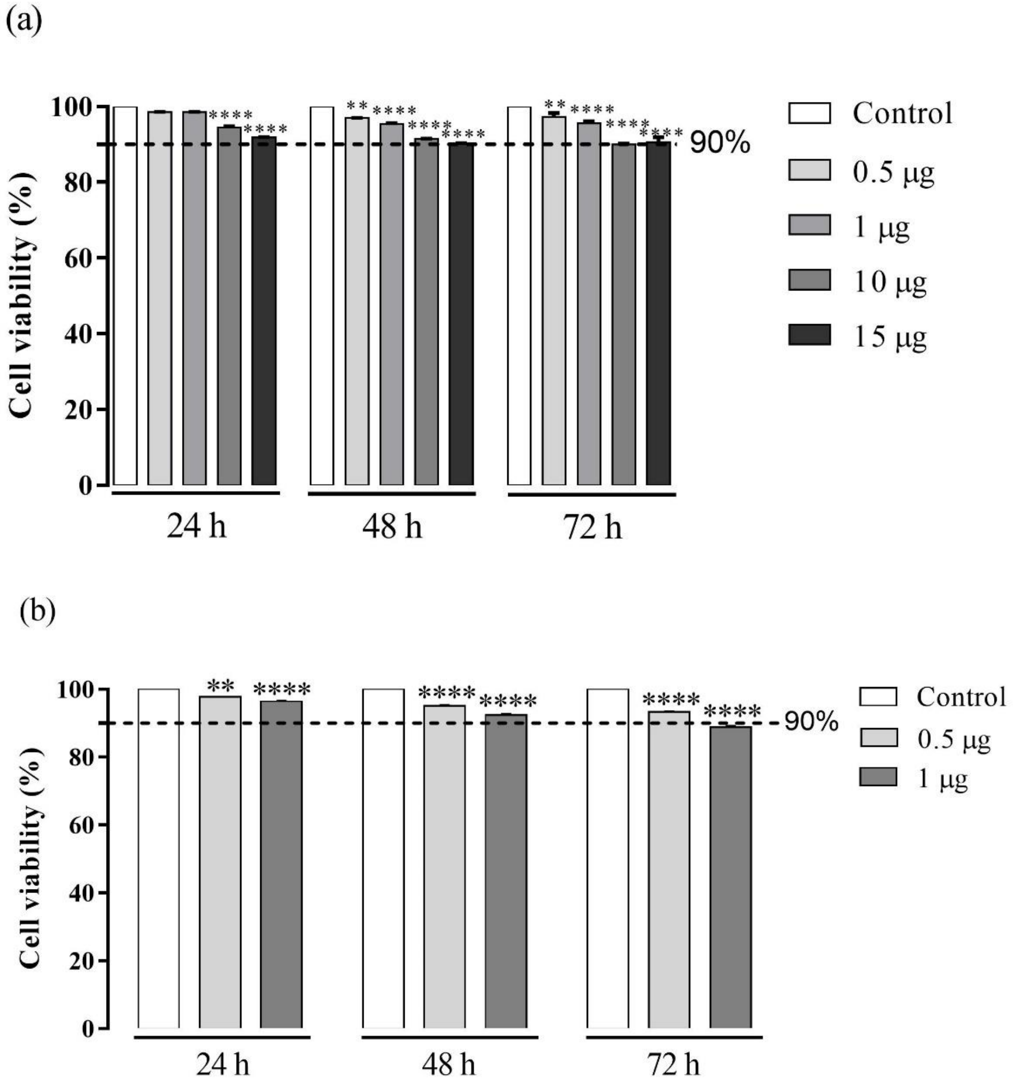

Figure S2. Cell cytotoxicity. Differentiated THP-1 macrophages was treated with 0.5–15 μg/well of BaV or with 0.5 and 1 μg/well Kn-Ba for 24 h, 48 h, and 72 h, and cell viability was evaluated by the quantification of lactate dehydrogenase (LDH) enzyme in cell-free supernatants. Results expressed as mean of duplicates ± SEM and analyzed by one-way ANOVA followed by Tukey's post-test. (*) Significant difference in relation to the respective control. $p < 0.01$ (**), and $p < 0.0001$ (****).
